# Opening the Conversation: study protocol for a Phase III trial to evaluate a couple-based intervention to reduce reproductive and sexual distress among young adult breast and gynecologic cancer survivor couples

**DOI:** 10.1186/s13063-022-06665-3

**Published:** 2022-09-02

**Authors:** Jessica R. Gorman, Karen S. Lyons, S. Marie Harvey, Chiara Acquati, John M. Salsman, Deborah A. Kashy, Julia H. Drizin, Ellie Smith, Lisa M. Flexner, Brandon Hayes-Lattin, Jennifer B. Reese

**Affiliations:** 1 College of Public Health & Human Sciences, School of Social & Behavioral Health Sciences, Oregon State University, 2250 SW Jefferson Way, Corvallis, OR 97330 USA; 2 Connell School of Nursing, Boston College, 140 Commonwealth Ave, Chestnut Hill, MA 02467 USA; 3 Graduate College of Social Work, University of Houston, 3511 Cullen Blvd, Houston, TX 77204-4013 USA; 4 College of Medicine, Department of Clinical Sciences, University of Houston, 4349 Martin Luther King Blvd, Houston, TX 77004 USA; 5 Department of Health Disparities Research, The University of Texas MD Anderson Cancer Center, 1515 Holcombe Blvd, Houston, TX 77030 USA; 6 Department of Social Sciences & Health Policy, Wake Forest School of Medicine, Medical Center Blvd, Winston Salem, NC 27157 USA; 7 Wake Forest Baptist Comprehensive Cancer Center, Medical Center Blvd, Winston Salem, NC 27157 USA; 8 Department of Psychology, Michigan State University, 316 Physics Road, East Lansing, MI 48824 USA; 9 Doctor of Physical Therapy Program, Oregon State University- Cascades, 1500 SW Chandler Ave, OR 97702 Bend, USA; 10 School of Medicine, Oregon Health & Sciences University, 3266 SW Research Dr, Portland, OR 97239 USA; 11 OHSU Knight Cancer Institute, 3485 S Bond Ave, Portland, OR 97239 USA; 12 Cancer Prevention and Control Program, Fox Chase Cancer Center, 333 Cottman Ave, Philadelphia, PA 19111 USA

**Keywords:** Breast cancer, Gynecologic cancer, Young adult, Reproductive health, Sexual health, Randomized controlled trial

## Abstract

**Background:**

Reproductive and sexual health (RSH) concerns are common and distressing for young adults diagnosed with breast and gynecologic cancer and their partners. This study evaluates the efficacy of a virtual couple-based intervention called Opening the Conversation (OC). The OC intervention is grounded in theory and evidence-based practice and was adapted to improve coping and communication specifically in relation to RSH concerns after cancer.

**Methods:**

This Phase III trial is conducted in a fully remote setting and enrolls young adult couples (current age 18–44 years) with a history of breast or gynecologic cancer (stage 1–4, diagnosed under age 40) within the past 6 months to 5 years. Eligible dyads are recruited from across the USA. The target sample size is 100 couples. Dyads are randomly assigned to receive either the 5-session OC intervention or a 4-session active control intervention (Side by Side). The primary outcomes are change in reproductive distress and sexual distress. Secondary outcomes include communication about reproductive concerns, communication about sexual concerns, depressive symptoms, sexual function, relationship quality, relationship intimacy, sexual satisfaction, self-efficacy to communicate about sex and intimacy, and quality of life. An exploratory aim examines whether dyadic coping and communication quality mediate intervention effects on survivors’ and partners’ reproductive distress or sexual distress. Self-report outcome measures are assessed for both groups at baseline (T1), 2 weeks post-treatment (T2), and 3 months post-treatment (T3).

**Discussion:**

Despite the importance of RSH for quality of life for young adult cancer survivors and their partners, evidence-based interventions that help couples navigate RSH concerns are lacking. This randomized controlled trial will determine the efficacy of a novel couple-based intervention to reduce distress related to RSH concerns for younger couples after breast or gynecologic cancer, in comparison to an active control intervention.

**Trial registration:**

ClinicalTrials.gov NCT04806724. Registered on Mar 19, 2021.

**Supplementary Information:**

The online version contains supplementary material available at 10.1186/s13063-022-06665-3.

## Administrative information

**Table:** 

Title	Opening the conversation: study protocol for a Phase III trial to evaluate a couple-based intervention to reduce reproductive and sexual distress among young adult breast and gynecologic cancer survivor couples
Trial registration	ClinicalTrials.gov, NCT04806724. Registered on Mar 19, 2021
Protocol version	Version 1 dated 05/11/2021
Funding	American Cancer Society RSG-19-123-01-CPPB
Author affiliations	Jessica R. Gorman, Oregon State University; Karen S. Lyons, Boston College; S. Marie Harvey, Oregon State University; Chiara Acquati, University of Houston; John M. Salsman, Wake Forest Baptist Comprehensive Cancer Center; Deborah A Kashy, Michigan State University; Julia H. Drizin, Oregon State University; Ellie Smith, Oregon State University; Lisa M. Flexner, Oregon State University-Cascades; Brandon Hayes-Lattin, OHSU Knight Cancer Institute; Jennifer B Reese, Fox Chase Cancer Center
Name and contact information for the trial sponsor	American Cancer Society, Inc. Extramural Discovery Science Department Email: grants@cancer.org Phone: 404-329-7558
Role of sponsor	N/A

## Background and rationale

Breast and gynecologic cancers (BGC) are among the most common cancers in young adults [1]. Survival rates in this population are high, as are the long-term burden of cancer and its treatment on physical and psychosocial outcomes [2–4]. Young BGC survivors are at particular risk for reproductive and sexual distress due to exposure to gonadotoxic treatment and/or treatment affecting pelvic nerves and organ structures as well as scarring, loss of breasts or feeling in the breast, and pain [5–7]. The reproductive and sexual health (RSH) consequences of cancer, including worries about infertility, body image, dating and intimate relationships, and sexual function [7–12], are among the most common and distressing aspects of survivorship for young BGC survivors and their partners [13–19]. Indeed, whereas sexual changes are common and distressing for patients of all ages, younger survivors and couples are at even greater risk of psychological distress due to such changes compared to their older counterparts [20–22]. In sum, RSH concerns can negatively affect intimate relationships, family building plans, and quality of life well after treatment ends [6, 12, 13].

Despite how important intimate relationships and reproductive health are in the quality of life for young adult survivors [23], there are no evidence-based interventions in place to help young BGC couples struggling with distress surrounding both reproductive and sexual health concerns. Additionally, most couple-based interventions were developed for heterosexual couples and have not focused on meeting the needs of LGBTQ+ survivors and their partners [24]. This is important because LGBTQ+ couples tend to be less satisfied with the care they receive, may not feel welcome in clinical and support group settings, and are at heightened risk for significant unmet survivorship care needs, particularly related to psychological and sexual health [25–28]. Available approaches also often neglect the needs of partners. Finally, supportive care to help couples with RSH concerns is not widely available, particularly in rural areas, and barriers accessing such care remain high [29, 30]. When available, services typically rely on in-person formats for delivery and involve therapists with specialized training, who are often constrained to serving clients in urban centers and large cancer centers.

Research demonstrates that psychosocial interventions targeted at couples can enhance dyadic coping (i.e., coping together as a team) and communication in the context of cancer [24, 31–33]. These skills are critical for managing distress and maintaining relationship health and quality of life [34–37]. Specifically, couple communication about reproductive and sexual health is increasingly recognized as an important predictor of relationship functioning [38–40]. Both partners can experience fear, uncertainty, and relationship difficulties due to fertility concerns [41, 42], suggesting that addressing such concerns for both partners would be important in an intervention. Regarding sexual concerns, the most effective approaches to addressing sexual health and reducing sexual distress after cancer actively engage partners [43–45]. Couple-focused interventions, therefore, represent a promising approach to managing RSH distress after cancer.

**Fig. 1:**
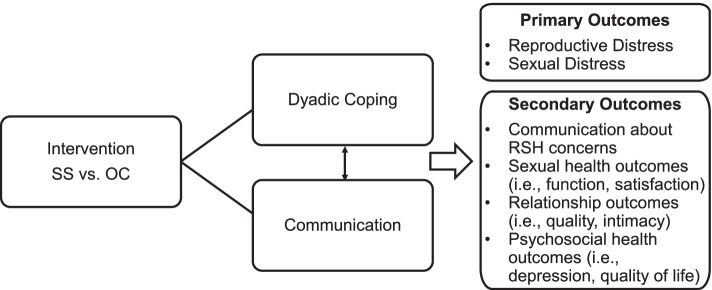
Study conceptual framework

Increasingly, psychosocial interventions for cancer survivors have been taking on a virtual format delivered via videoconference. Because virtual delivery of psychosocial interventions can remove barriers to access among those in less well-resourced cancer care settings and rural areas [46, 47], this format offers a promising approach to fill a gap in supportive care for young couples from all geographic areas faced with navigating life after cancer [9, 14]. Prior research points to the feasibility of a virtual intervention format [47], particularly for recruiting more ethnically and geographically diverse patients as well as less healthy patient populations [48]. The primary objective of this Phase III trial is to evaluate the efficacy of a virtual psychosocial intervention, Opening the Conversation (OC), to reduce RSH distress among young BGC survivors and their partners. OC was developed through systematically adapting an intervention initially designed to help couples cope with and communicate about cancer distress in general, called Side by Side (SS). OC was designed to help young couples cope together and communicate effectively about RSH concerns specifically. SS and OC are grounded in methods of cognitive behavioral therapy [34] and Bodenmann’s Systemic Transactional Model of dyadic coping [36, 49–53]. SS was previously modified from CanCOPE [54] in a pilot trial [55], then adapted specifically for virtual delivery with young adult BGC survivor couples in preparation for the current study [56]. In a prior in-person randomized controlled trial of SS, conducted in Germany with 72 heterosexual couples (female survivors of breast or gynecologic cancer and their partners, median age 52 years), couples receiving the SS intervention reported less avoidance in dealing with cancer, more posttraumatic growth, better communication quality, and better dyadic coping than those in an attention control condition [49]. A virtual version of SS, which focuses on general cancer-related distress, serves as the comparison condition to the OC intervention. The study conceptual framework is shown in Fig. 1. This manuscript describes the study protocol for a Phase III randomized controlled trial comparing two parallel conditions, OC and SS.

### Objectives

The specific aims of this study are:

1. To evaluate whether the OC intervention leads to significantly greater improvement in reproductive distress and sexual distress reported by BGC survivors and their partners, as compared to the SS intervention (Aim 1a, primary outcomes)
2. To evaluate whether the OC intervention leads to significantly great improvement in cancer survivors’ and their partners’ relationship (relationship quality, relationship intimacy, sexual function, sexual satisfaction, self-efficacy to communicate about sex and intimacy) and psychosocial health (depressive symptoms, quality of life) outcomes, as compared to the SS intervention (Aim 1a, secondary outcomes)
3. In exploratory analyses, we will evaluate whether dyadic coping and communication quality mediate intervention effects on survivors’ and partners’ reproductive distress or sexual distress. (Aim 1b)

## Methods

### Trial design

**Fig. 2:**
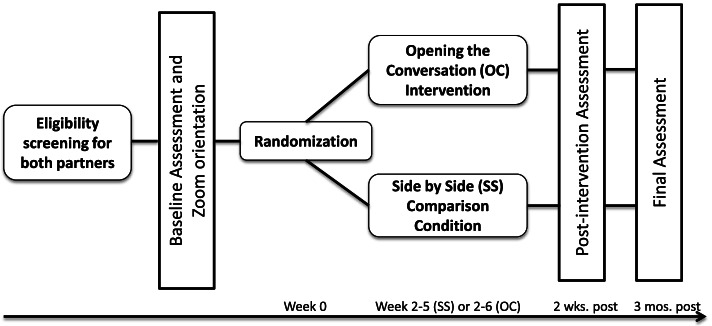
Study flow diagram

The study will enroll 100 dyads (couples), each comprised of a breast or gynecological cancer survivor (diagnosed between 18 and 39 years of age) and their identified intimate/romantic partner. This is a two-group randomized controlled trial design with one pre-test and two post-test quantitative assessments. Dyads will be randomized to either OC or SS, with equal allocation to each condition. OC includes 5 weekly sessions, 90 min each. SS includes 4 weekly sessions, 90 min each. Both OC and SS are delivered remotely by a trained interventionist to participants across the USA. Web-based assessments are collected at baseline (T1), 2 weeks post-intervention (T2), and 3-month follow-up (T3). This study protocol has followed the Standard Protocol Items: Recommendations for Interventional Trials (SPIRIT) statement guidelines (See SPIRIT Checklist) [57]. The study will be conducted and reported according to the Consolidated Standards of Reporting Trials (CONSORT) criteria [58]. The study has been approved by the institutional review board (IRB) at Oregon State University (IRB 7621). The study flow is shown in Fig. 2.

### Eligibility criteria

Cancer survivor participants are eligible if they meet the following criteria: (1) cancer diagnosis between the ages of 18 and 39, (2) current age 1844, (3) cancer diagnosis between 6 months and 5 years prior to enrollment date, (4) diagnosed with breast or gynecologic cancer, (5) self-reported cancer stages 1–4 at time of diagnosis, (6) self-reported ability to participate in a virtual intervention, (7) self-reported committed intimate partner who is willing to participate (any gender/sexual orientation), (8) English speaking, and (9) high-speed internet access via a smart phone, tablet, and/or computer.

Partners are eligible if they meet the following criteria: (1) age 18 or older, (2) English speaking, (3) self-reported ability to participate in a virtual intervention via videoconference, and (4) high-speed internet access via a smart phone, tablet, and/or computer.

Survivors and partners are excluded if either partner does not meet the eligibility criteria. Both partners must enroll in order to participate.

### Procedures

#### Recruitment and informed consent

Study recruitment is multi-pronged in that it includes a range of sources of participant identification, including study advertisements online via social media platforms, outreach through community partners, and letters mailed to potentially eligible cancer survivors in the Oregon State Cancer Registry (OSCaR). This strategy was selected both to facilitate achieving recruitment targets and to reach a diverse sample of dyads nationwide in light of the differences in survivors found through these sources.

Consent is obtained electronically after both partners complete screening procedures and potential participants can discuss their questions with a member of the research staff. Once both members of the dyad have completed consent, the dyad is considered to be consented. Enrollment is determined at randomization, after both partners complete the baseline assessment.

#### Data collection

Quantitative data are collected using Research Electronic Data Capture (REDCap). Each member of the dyad will complete separate surveys via REDCap at T1, T2, and T3, which are expected to take approximately 30 min each. Individual participants can receive compensation for the study of up to $30 in gift cards, or $60 in gift cards per dyad for completing all assessments. Strategies to increase retention include web-based surveys, flexible scheduling of intervention sessions, and sending email reminders. If either member of the dyad is unable or chooses not to continue participating in the intervention, both may be asked if they would be willing to remain in the study for data collection only and we will attempt to collect data as though they had participated in the assigned intervention.

Study staff complete training in human subject protection, secure data management, and protocols and procedures. All assessments occur in REDCap, which could reduce error related to manual data entry. Routine data checks are in place to check for data quality. Data safety and monitoring will occur in monthly investigator meetings. The group will monitor recruitment, enrollment, data collection, procedures, and any adverse events or protocol deviations (tracked and reported as required by the IRB). Data security is maintained through password-protected files, restricted access to datasets, and de-identification of data when possible.

#### Randomization

The study uses 1:1 blocked randomization (groups of 4), stratified by cancer type (breast versus gynecologic) and sexual orientation reported by the cancer survivor (heterosexual versus other). The study statistician generates the randomization sequence, and assignment to condition occurs via REDCap after the dyad has completed both the baseline assessment and videoconference orientation call with study staff. Couples are notified of their intervention assignment after scheduling their first session with the study interventionist. At that time, they receive both a mailed version and link to an electronic version of the participant materials. As with most behavioral interventions, the study interventionist, intervention supervisor, and participants are not masked to condition. However, although participants know whether they are receiving OC or SS, they are not told which of these is the experimental condition. The condition is masked to the PI, biostatistician, and research assistants who will evaluate quantitative outcome data. Quantitative outcome data is also collected via REDCap to minimize contact with participants while collecting study data. Should a review of adverse events reveal significant between-group differences that could warrant unmasking of the study conditions by the study biostatistician.

### Interventions

#### Intervention overview

Participants in both conditions receive a manualized virtual psychosocial intervention, either OC or SS. The OC intervention includes 5 weekly sessions, and the SS intervention (comparison) includes 4 weekly sessions. Both interventions are delivered by a trained interventionist following a standard intervention protocol and integrating participant handouts with educational material and skill-building exercises. Each session is approximately 90 min. All sessions are audio-recorded. There are no restrictions on receipt of concomitant care participants may receive while enrolled in the study.

#### Interventionist training and adherence

Interventionists have a master’s degree in a mental health field (e.g., social work, psychology) and experience working clinically with cancer survivors and their partners/family members. Training includes certification in delivery of both intervention conditions by two study investigators (JG, JR). Training is done remotely via a workshop for which trainees are asked to review materials ahead of time, including the study protocol (interventionist manuals, participant materials) and background readings on relevant topics such as young adult breast/gynecologic cancer, RSH after cancer, couple-based intervention, and theoretical foundations. The remote workshop includes a review of the protocols, key concepts from the required readings, specific skills, and discussions. This is followed by a mock session delivery with feedback. Interventionists complete a full test case with each condition and receive feedback from the supervisor (Co-I Reese, who is a licensed psychologist and has experience in delivering and supervising couples’ interventions in cancer). Training also emphasizes the importance of fidelity to the interventionist manuals, and strategies for developing and maintaining rapport and engagement via videoconference. Interventionists complete reports after each session to document adherence to the manual, to indicate whether there were any unplanned diversions, and to document any challenges faced. Supervision occurs throughout the study and includes review of sessions, audio recordings, and interventionist reports of adherence. Supervision also includes regular group meetings to discuss cases and problem solve. A trained independent reviewer not involved in intervention delivery will randomly select and review 15% of sessions to independently document adherence.

#### Study arms

##### Opening the Conversation: experimental intervention group

The intervention group, OC, receives 5 weekly sessions with a trained interventionist. Session content is outlined in Table 1. Couples learn coping and communication strategies that increase their support for one another, particularly related to reproductive and sexual distress. The comparison arm, SS, provided a foundation for the development of OC, but OC was adapted to specifically address coping and communication about RSH concerns after cancer. Modifications were based on an iterative process of interviews with couples and stakeholder feedback. Briefly, these included new educational material about RSH-related concerns after cancer for both partners, integration of opportunity to discuss RSH topics across sessions, and the addition of a fifth session to give couples the opportunity to use new skills learned to discuss an RSH topic of their choice. Details about the systematic adaptation process and resulting changes are available elsewhere [56].

**Table 1:** Opening the Conversation (OC) and Side by Side (SS) session summaries

Session	Opening the Conversation	Side by Side
1	• Introduction and overview • Cancer’s impact on relationships and RSH • Introduce educational materials on RSH concerns after cancer • Introduce supportive communication, supportive behavior, mindfulness	• Introduction and overview • Cancer’s impact on relationships • Introduce supportive communication, supportive behavior, mindfulness
2	• Stressors and stress response, with focus on RSH • Coping strategies and self-talk, with examples related to RSH • Practice supportive communication, focusing on an RSH stressor	• Stressors and stress response, with focus on cancer experience • Coping strategies and self-talk • Practice supportive communication, focusing on a cancer-related stressor
3	• Practice supportive communication, focusing on an RSH stressor • Communication with family/friends, with examples related to RSH • Patient advocacy and healthcare provider communication about RSH concerns	• Practice supportive communication • Communication with family/friends
4	• Sexual health concerns, emotional and physical intimacy • Communication exercise to build shared understanding about intimacy • Identifying and scheduling activities to support intimacy • Setting goals to support intimacy	• Emotional intimacy • Making time for activities to support relationship • Explore positive aspects of your cancer experience • Setting goals to support relationship
5	• Reproductive health and family building concerns • Practice supportive communication with RSH topic • Skill review • Setting goals to support relationship	None

##### Side by Side: active comparison group

The comparison group, SS, receives 4 weekly sessions with a trained interventionist. Session content is outlined in Table 1. Couples learn coping and communication strategies that increase their support for one another as they navigate cancer-related stressors in general. SS includes a discussion and exercise focused on emotional, but not physical, aspects of intimacy. SS was selected as a comparator because of its grounding in theory and evidence-based practice along with its demonstrated effectiveness for improving relationship and psychosocial outcomes among BGC survivors [34, 36, 49]. Original content was updated for virtual delivery to our intended audience, including use of inclusive language for LGBTQ+ couples.

### Measures

The outcome measures used are reliable and valid with cancer survivors. We selected short form versions, where available, to reduce participant burden. All outcome measures are assessed at 3 time points: baseline (immediately prior to intervention), 2 weeks after the last session, and 3 months after the last session. All outcome measures and assessment instruments are listed in Table 2.

**Table 2:** Schedule of enrollment, interventions, and assessments

	Enrollment eligibility T0	Allocation baseline T1	Post-allocation			
			Sessions 1–4	Sessions 1–5	T2 2 weeks	T3 3 months
ENROLLMENT						
Eligibility screen	X					
Informed consent	X					
Allocation		X				
INTERVENTIONS						
OC intervention				X		
SS intervention			X			
ASSESSMENTS						
Primary outcomes						
Reproductive distress Fertility Problem Inventory (FPI)-Relationship Concern Domain		X			X	X
Sexual distress Sexual and Relationship Distress Scale (SaRDS)		X			X	X
Secondary outcomes						
Relationship functioning Dyadic Adjustment Scale (DAS-7)		X			X	X
Relationship intimacy Miller Social Intimacy Scale (MSIS)		X			X	X
Communication about reproductive concerns Couples’ Illness Communication Scale (CSIS)		X			X	X
Communication about sexual concerns Couples’ Illness Communication Scale (CSIS)		X			X	X
Sexual function (female) Female Sexual Function Index (FSFI)		X			X	X
Sexual function (male) International Index of Erectile Function (IIEF)		X			X	X
Sexual satisfaction General Measure of Sexual Satisfaction (GMSEX)		X			X	X
Depressive symptoms Patient Health Questionnaire Depression Scale (PHQ-8)		X			X	X
Health-related quality of life PROMIS Global 10 v1.2		X			X	X
Self-efficacy^a^ Self-Efficacy to Communicate about Sex and Intimacy (SECSI)		X			X	X
Mediating variables						
Dyadic coping	Dyadic Coping Inventory (DCI)	X			X	X
Communication quality	Communication Patterns Questionnaire- Short Form (CPQ-SF)	X			X	X

^a^Cancer survivor-only measure

Intervention acceptability and appropriateness and interventionist assessments are measured 2 weeks after the last session. Two process measures (perceived usefulness and session-specific evaluations) are measured during the intervention.

#### Primary outcome measures

##### Reproductive distress

Reproductive distress is measured using the Fertility Problem Inventory (FPI)-Relationship Concern Domain. This measure consists of 10 items assessing concerns about infertility’s impact on the relationship. Higher scores indicate a higher level of distress [59–61].

##### Sexual distress

Sexual and relationship distress is measured with the Sexual and Relationship Distress Scale (SaRDS), a 30-item, multidimensional scale that measures relationship distress within the context of sexual difficulties [62]. Item responses are summed to create a total summary score, with higher scores indicating more sexual distress.

#### Secondary outcome measures

##### Communication about reproductive concerns

Communication about reproductive concerns is assessed via the Couples’ Illness Communication Scale, a four-item scale adapted to measure couple communication about reproductive/fertility concerns [63]. Item responses are summed to create a total score, with higher scores indicating better reproductive-related couple communication.

##### Communication about sexual concerns

Communication about sexual concerns is also assessed via the Couples’ Illness Communication Scale [63], adapted to measure couple communication about sexual concerns. Higher scores indicate better sex-related couple communication.

##### Depressive symptoms

Depression is measured using the Patient Health Questionnaire Depression Scale (PHQ-8), an eight-item scale assessing depression severity [64, 65]. Higher scores reflect higher depressive symptoms.

##### Female sexual function

Female sexual function is measured via the Female Sexual Function Index (FSFI), a 19-item, multidimensional scale assessing the following domains of female sexual function including desire, arousal, lubrication, orgasm, pain, and satisfaction [66, 67]. Subscale scores are summed to create a total score, with higher scores indicating better functioning.

##### Male sexual function

Male sexual function is measured via the International Index of Erectile Function (IIEF), a 15-item, multidimensional scale assessing various domains of male sexual function [68, 69]. Item responses within each domain are summed to create subscale scores, and subscale scores are summed to create a total score, with higher scores indicating better functioning.

##### Relationship quality

Relationship functioning is measured with the Dyadic Adjustment Scale (DAS-7), a seven-item scale measuring relationship functioning [70]. Responses are summed to create a total score, with higher scores reflecting better relationship functioning [70–72].

##### Relationship intimacy

Intimacy is measured using the Miller Social Intimacy Scale, a 17-item scale that measures the level of intimacy currently experienced in an interpersonal relationship [73]. Responses are summed, with higher scores reflecting higher levels of intimacy.

##### Sexual satisfaction

Sexual satisfaction is measured with the General Measure of Sexual Satisfaction (GMSEX), a five-item scale assessing the sexual relationship, with higher scores indicating greater sexual satisfaction [74, 75].

##### Self-efficacy to communicate about sex and intimacy

Self-efficacy is measured for cancer survivors only using the Self-Efficacy to Communicate about Sex and Intimacy (SECSI) scale. This 10-item measure assesses cancer survivors’ self-efficacy for sexual health communication over the last month, with higher scores indicating higher self-efficacy [65].

##### Quality of life

Quality of life is assessed via the PROMIS (Patient-Reported Outcome Measurement Information Systems) Global 10-item scale, which measures aspects of physical and mental health. Scores are standardized to the general population [76, 77].

#### Intervention mediators

##### Dyadic coping

Dyadic coping is measured with the Dyadic Coping Inventory, a 37-item scale measuring perceived communication and dyadic coping among couples. This scale consists of several subscales measuring supportive, delegated, negative, and joint coping. Responses to each item are summed to create a total score, with higher scores indicating higher relationship quality [78, 79].

##### Communication quality

Communication quality is measured using the Communication Patterns Questionnaire-Short Form (CPQ-SF), an 11-item self-assessment measuring perceptions of relationship interactions. The CPQ-SF assesses different patterns of communication (non-constructive communication and constructive communication), with higher scores indicating more use of that communication pattern during conflict [80].

#### Demographics, cancer, and reproductive history

Socio-demographic characteristics including age at baseline, race, ethnicity, sexual orientation, gender identity, education level, number of children, and employment status are collected via self-report. Relationship, cancer, and reproductive characteristics are also assessed by self-report, including relationship status and duration, desire for children, cancer type, stage, and treatments, time since diagnosis, and reproductive history.

#### Acceptability, appropriateness, and feasibility

Intervention acceptability and appropriateness are assessed via AIM/FIM subscales [81] and a counselor assessment form. Perceived usefulness is assessed after the first session with items asking how logical the program seemed, how helpful they think the program will be, and how competent they believe the counselor is. Each week, participants are asked to assess the extent to which they interacted with and understood the program material.

### Statistical plan

We will compare recruitment, retention, satisfaction, and completion of assessments for the intervention and comparison group. Our approach to recruitment and retention minimizes missing data by monitoring and actively following up with reminders for survey completion, and by incentivizing participation. Initial analyses will examine characteristics of non-completers. As recommended by Lang & Little [82], multiple imputation (using 50 imputed samples) will be used to impute missing values for analyses using multilevel modeling (MLM) and full information maximum likelihood (FIML) will be used for mediation analyses conducted within a structural equation modeling framework.

Basic descriptive data analyses will be conducted to examine means, standard deviations, and correlations among the study measures. Normality of outcomes will be assessed, and data transformations will be applied as needed.

Multilevel modeling (MLM; SPSS Version 27) with REML will be used to test Aim 1a for both the primary (reproductive and sexual distress) and secondary outcomes (relationship outcomes and psychosocial health outcomes). These models will examine mean differences in outcomes between OC and SS over time (baseline, 2 weeks, 3 months) and across role (i.e., survivor versus partner) for outcomes measured from both partners. In our primary analyses, time, treatment, and role will be treated as categorical, and models will include all main effects and interactions among these variables. Evidence of treatment efficacy will be assessed by the interaction between time and treatment as we expect no treatment differences at baseline, but we expect to see such differences after the intervention. These models will include random intercepts for survivors and partners, as well as the correlation between the intercepts (i.e., if a survivor is high in average distress across time, is the partner high as well?). They will also include a time-specific correlation between the partners’ residuals (i.e., if a survivor is especially distressed at a particular time point, is the partner especially distressed at that time as well?). In addition to being able to model the interdependence between survivors and partners, the MLM approach has the advantage that it does not delete participants with missing data at some time points and so this analysis utilizes all available data. Outcomes measured only for survivors or only for partners will also be analyzed using MLM, but in this case role will not be included as a predictor and the random effects will include only a random intercept and residual variance.

Covariates to be assessed include age, time since diagnosis, cancer stage, metastatic status, number of children, relationship duration, income, race, ethnicity, and sexual orientation. To determine which covariates should be included in the analyses, preliminary analyses will be conducted in which each outcome is predicted by the full set of qualifying covariates. Only those covariates that predict at least one outcome significantly will be included in the analyses. Once the set of covariates are determined in this way, the same set of covariates will be included in all analyses.

Exploratory Aim 1b posits a mediational model in which the effects of the intervention on both partners’ primary outcomes are mediated by both partners’ dyadic coping and communication. To examine mediation, the statistical package MPLUS will be used to estimate and test the indirect effects of the treatment on 3-month outcomes via the patient’s and partner’s dyadic coping and communication measured 2 weeks after the intervention. The same set of covariates used in tests of Aim 1a will be included.

#### Sample size estimate/ power calculations

A sensitivity power analysis was conducted using the program G*Power and anticipating an initial sample of 100 couples (*N* = 200 individuals), and an attrition rate of 20% at the final assessment. Given that the design has two levels of the treatment and three assessments, for outcomes that are assessed on only survivors (or only partners), the study has 90% power to detect small to moderate effects (i.e., *d* = .32) if there is no attrition. With the expected 20% attrition, the study has 80% power to detect effects greater than *d* = .36. Because both partners will complete most of the same outcomes, for these outcomes the effective sample size is larger than the number of dyads but smaller than the number of individuals. Specifically, assuming a cross-dyad correlation of *r* = .4 (e.g., the correlation between the two partners’ distress), the effective sample size to test the treatment by time interaction is 142 without attrition and 114 with attrition. Thus, for these outcomes, without attrition the study will have 90% power to detect effects of *d* > .27 and with attrition it will have 90% power to detect effects of *d* > .30.

### Ethical aspects

This trial was approved by the Oregon State University Human Research Protections Program (Protocol 7621). All investigators and research staff have been trained in principles of ethical conduct of human subject research. Study participants complete informed consent procedures. Financial compensation ($10 per survey) is similar to that in other couple-based intervention studies and intended to express appreciation to participants for their time. As a minimal risk study, a Data and Safety Monitoring Board was not needed. Investigators meet monthly to monitor study progress and discuss data collection and study progress, including monitoring risks and benefits to participants. The study coordinator is responsible for tracking enrollment procedures and ensuring that data collection is complete. Adverse events are reported to the PI within 1 week and are summarized quarterly for the governing IRB. Protocol revisions will be approved by the governing IRB and reported to ClinicalTrials.gov (NCT04806724). Table 3 outlines trial registration data. In very rare cases where high distress is observed that is severe enough that continuing the study is judged to interfere with participants’ well-being, couples will be counseled to discontinue and provided resources and information encouraging follow-up care. As a minimal risk study, there are no provisions in place for ancillary, post-trial, or compensation for study-related harms.

**Table 3:** Trial registration data

Data category	Information
Primary registry and trial identifying number	ClinicalTrials.gov
Date of registration in primary registry	March 19, 2021
Secondary identifying numbers	None
Source(s) of monetary or material support	American Cancer Society
Primary sponsor	American Cancer Society
Secondary sponsor(s)	None
Contact for public queries	JG, PhD, MPH [Jessica.Gorman@oregonstate.edu]
Contact for scientific queries	JG, PhD, MPH [Jessica.Gorman@oregonstate.edu]
Public title	Opening the Conversation
Scientific title	Opening the Conversation for Couples With Reproductive Health Concerns
Countries of recruitment	USA
Health condition(s) or problem(s) studied	Reproductive and sexual distress after cancer
Intervention(s)	Experimental Intervention Group: Opening the Conversation intervention, 5 sessions Active Comparison Group: Side by Side intervention, 4 sessions
Key inclusion and exclusion criteria	Ages eligible for study: ≥18 years to 44 years (cancer survivor, partner 1) and ≥18 years (partner 2) Sexes eligible for study: all Accepts healthy volunteers: no
	Inclusion criteria (partner 1): cancer diagnosis between the ages of 18 and 39; current age 18–44; cancer diagnosis between 6 months and 5 years prior to enrollment date; diagnosed with breast or gynecologic cancer; self-reported cancer stages 1–4 at time of diagnosis; committed partner who is willing to participate Inclusion criteria (partner 2): age 18 or older
	Exclusion criteria: survivors and partners are excluded if either partner does not meet the eligibility criteria; both partners must enroll in order to participate.
Study type	Interventional
	Allocation: randomized intervention. Parallel assignment masking: double blind (investigator, outcomes assessor)
	Primary purpose: supportive care
	Phase III
Date of first enrolment	September 2021
Target sample size	100 dyads (200 individuals)
Recruitment status	Recruiting
Primary outcome(s)	Reproductive distress, Sexual distress (time frame: 3 months)
Key secondary outcomes	Communication about reproductive concerns, communication about sexual concerns, depressive symptoms, sexual function, relationship quality, relationship intimacy, sexual satisfaction, self-efficacy to communicate about sex and intimacy, and quality of life

## Discussion

Upon completion of this Phase III trial, we will have rigorously tested the efficacy of a novel theory-driven virtual intervention, called Opening the Conversation (OC), addressing a range of RSH concerns experienced by young BGC survivors and their partners, with attention to inclusivity for LGBTQ+ individuals. If efficacious, the OC intervention could help fill known gaps in care for young couples who are experiencing RSH-related distress [83–85]. We will also examine theoretically based mediators of OC intervention effects; these analyses are novel as there is little known about possible mechanisms underlying the effects of couple-based psychosocial interventions in cancer, and even less known about mechanisms of interventions focusing on RSH.

Novel aspects of OC include the focus on both reproductive and sexual concerns, which often co-occur for younger couples [85, 86], and inclusivity for LGBTQ+ couples. Furthermore, the intervention is grounded in proven cognitive behavioral therapy techniques [34], in valid theoretical models (i.e., Bodenmann’s Systematic Transactional Model of Dyadic Coping [51, 52]), and prior evidence-based interventions [50, 54, 87]. These aspects serve as important strengths of this intervention. Moreover, the OC intervention covers a broad range of RSH topics (i.e., contraception, fertility potential, pelvic health) and potential action steps (e.g., type of healthcare professional to talk to about the concern), making it well-suited to meeting the varied needs of BGC survivors and their partners. Finally, the intervention is designed for virtual delivery, which could increase reach and improve access for young couples [88, 94] and was preferred by couples in the formative research phase [35].

This study will be the first to evaluate the effect of a psychosocial intervention focused on reducing RSH distress in young cancer survivor couples. One strength of the study design is the use of an active comparison condition, SS, which has been previously tested and found to be effective in improving support and communication skills for couples experiencing cancer-related distress [49]. With the inclusion of this skills training content in the active control, we expect to see benefits for both OC and SS, but anticipate more significant reductions in RSH distress for those in the OC condition, where RSH-related issues are explicitly addressed. Furthermore, we designed the OC intervention with broader implementation and dissemination in mind, such as consideration of feasibility and practicality. We anticipate that planning an efficacy trial with later-stage implementation and dissemination in mind may help reduce barriers to future implementation in practice to facilitate adoption and use of the intervention in real-life practice [95, 96]. One key example of an effort taken to enhance real-world adoptability of OC is the use of master’s level interventionists, rather than PhD level interventionists, which aligns with the practice in most cancer centers. We also developed standardized training materials for study interventionists; if OC is found to be effective, these materials could be used to train other mental health professionals with a similar background as part of future dissemination efforts. Additionally, we include assessment of perceived feasibility and acceptability of the intervention, with the goal of identifying further modifications to facilitate its future implementation.

The current study design also has limitations. First, only individuals with a committed partner are able to participate, which necessarily excludes unpartnered individuals. Future studies should assess the needs of women with breast or gynecologic cancer with RSH concerns who are not partnered in order to design appropriate interventions for this population, as their needs may differ from those of partnered women. Second, because the intervention’s focus is on improvements at the individual and couple levels, it does not address other important social or structural determinants such as access to RSH care after cancer. Additional multilevel strategies may complement and reinforce couple-based approaches to improving equity in RSH care after cancer. Finally, although we purposefully engaged LGBTQ+ couples in the adaptation process for OC [56], based on prior research experience, and results of similar trials that do not limit enrollment by sexual orientation [97], it is likely that most participants will be heterosexual and cisgender. Although we would ideally be able to examine outcomes and potential differences based on sexual orientation and gender, we may have too small a sample size of LGBTQ+ couples to accomplish this. Thus, future studies should consider focusing efforts on these specific populations. Finally, the focus on BGC cancer couples specifically excludes young couples with other types of cancer who experience RSH concerns [5, 7, 98]. If OC proves effective, further adaptations could be made for other cancer populations. Despite these limitations, results of the study will contribute significantly to our understanding of the role of psychosocial interventions for reducing RSH distress after cancer.

A large number of young BGC couples experience RSH concerns after cancer, and these concerns often go unaddressed. Evidence-based interventions are needed to fill this gap in supportive care in survivorship. Results are expected to determine whether the OC intervention improves reproductive distress and sexual distress reported by BGC survivors and their partners, as compared to the SS intervention. It is also expected to yield important information about mechanisms of change underlying intervention-related improvement in outcomes and could document the needs of subgroups of survivors and partners that would help in further targeting the intervention. Upon completion of this trial, the study team will be prepared to consider next steps, which may include expanding the intervention to multiple sites to explore implementation, dissemination, and strategies for integration into comprehensive survivorship care and further intervention adaptation and evaluation with other cancer survivor populations.

### Dissemination plans

The findings of this trial will be disseminated to researchers and the public through the study’s entry on ClinicalTrials.gov, through publication in peer-reviewed journals, and through presentation of the findings to the scientific community at scientific conferences. To reach a wider audience, summary results will also be shared widely with participants, advocacy groups, and community partners such as via social media platforms.

## Trial status

This trial is actively recruiting participants. Recruitment began in September 2021 and is expected to continue through 2023. This manuscript describes the study protocol dated 05/11/2021.

## Supplementary Information

**Additional file 1.** Model research consent form.

## Data Availability

De-identified quantitative data will be stored electronically without any personal identifying information for open access. Data will be stripped of indirect and direct identifying information before sharing. All study data and relevant materials from the trial described in this manuscript will be retained and archived by the primary study site for a minimum of 3 years after study completion.

## Abbreviations

BGC: Breast and gynecologic cancer
CPQ-SF: Communication Patterns Questionnaire-Short Form
CSIS: Couples’ Illness Communication Scale
DAS-7: Dyadic Adjustment Scale-7 item
DCI: Dyadic Coping Inventory
FIML: Full information maximum likelihood
FSFI: Female Sexual Function Index
FPI: Fertility Problem Inventory
GMSEX: General Measure of Sexual Satisfaction
IIEF: International Index of Erectile Function
MLM: Multilevel modeling
MSIS: Miller Social Intimacy Scale
OC: Opening the Conversation
PHQ-8: Patient Health Questionnaire-8 item
PROMIS: Patient-Reported Outcome Measurement Information System
REML: Restricted maximum likelihood
RSH: Reproductive and Sexual Health
SaRDS: Sexual and Relationship Distress Scale
SECSI: Self-Efficacy to Communicate about Sex and Intimacy
SS: Side by Side
