# Supplementary material for: Opening the Conversation: study protocol for a Phase III trial to evaluate a couple-based intervention to reduce reproductive and sexual distress among young adult breast and gynecologic cancer survivor couples
Source: Trials. 2022 Sep 2;23:730. doi: 10.1186/s13063-022-06665-3 (PMC9438271; doi:10.1186/s13063-022-06665-3)
Supplement: Supplementary file 1 — Additional file 1. Model research consent form. [file 13063_2022_6665_MOESM1_ESM.doc]

Additional file 1. MODEL RESEARCH CONSENT FORM

**Project Title: Opening the Conversation**

**Principal Investigator:** Jessica Gorman, PhD

**Co-Investigators:** Marie Harvey, DrPH; Karen Lyons, PhD; Jennifer Reese, PhD; Brandon Hayes-Lattin, MD; Deborah Kashy, PhD; John Salsman, PhD; Chiara Acquati, PhD

**Sponsor:** American Cancer Society

**Version: 1.0**

We are inviting you to take part in a research study.

This form contains information you will need to help you decide whether to be in this research study or not. Please read the form carefully and ask the study team members questions about anything that is not clear.

**Purpose:** We are doing this research to test the helpfulness of two programs designed to teach breast and gynecologic cancer survivors and their partners ways to communicate and cope with physical, emotional, and relationship challenges after cancer. We are asking you to take part in this study because you or your partner/spouse has been diagnosed with breast or gynecologic cancer under age 40. The sponsor of this study is the **American Cancer Society**.

**Activities:** If you agree to be in this study, you and your partner/spouse will be randomly assigned to one of two programs. This means you will be put into a program by chance, like flipping a coin. Neither you nor the study team will be able to choose the program group you are assigned to. The program groups will be equal in number of participants.

Program 1: We will ask you and your partner/spouse to take part in 5 private videoconference sessions (via Zoom) together with our trained counselor. You and your partner will receive worksheets and educational materials about cancer, communication, coping as a couple, and mindfulness exercises, with a focus on reproductive concerns and intimacy after cancer. We will ask you both to read materials, take part in study activities, and answer brief session evaluation surveys between each session.

Program 2: We will ask you and your partner/spouse to take part in 4 private videoconference sessions (via Zoom) together with our trained counselor. You and your partner/spouse will receive worksheets and educational materials about cancer, communication, coping as a couple, and mindfulness exercises, with a focus on your cancer journey. We will ask you both to read materials, take part in study activities, and answer brief session evaluation surveys between each session.

For both programs, each weekly session will last about 1.5 hours and will be audio recorded. We will schedule these sessions at a time that is good for you. They will be spaced about a week apart. In the case that sessions need to be rescheduled or scheduled further apart, we ask that couples complete all sessions within 8 weeks.

We are asking you to fill out 3 surveys. You will do these online using a link we give only to you. The surveys ask things about you, such as your age, race, ethnicity, and sexual orientation, and about aspects of your physical, emotional, relationship, reproductive, and sexual health. You do not have to answer any questions you do not want to. You will spend about 30 minutes filling out each survey. We will ask you do all surveys separately from your partner.

We may also ask you and your partner/spouse to individually participate in a telephone interview one month after the last session. You do not have to answer any questions you do not want to. This interview will last about 60 minutes.

After couples have completed all of the study activities, we will send you the handouts from the program that you did not participate in and a link to connect to a private Facebook group if you are interested in connecting with other survivors/partners who participated.

**Time:** For most people, it will take about 5-6 months to complete the whole study.

**Voluntary:** Being in this study is your choice. You can agree to be in the study now and change your mind later. You may choose not to answer any questions or participate in any aspect of the study that you are uncomfortable with.

**Storage and future use of data:** We will store de-identified data collected during this study

indefinitely. Data will be stored electronically without any personal identifying information. Keeping data will allow researchers to continue to answer new research questions that may arise and to compare the results of this study to those of future studies.

**Risks:** There are no known risks or side effects expected to happen in this study. There is a possible risk of loss of privacy. We will make every effort to keep your information private, but this cannot be guaranteed. You could become distressed when answering questions related to sensitive topics such depression, fertility, or sexual problems. It is possible that you or your partner may feel some added distress from talking about these issues while taking part in this study. You do not have to answer any question you do not want to answer and you may take a break at any time during the study. You may also stop being in this study at any time.

**Benefit:** We do not know if you will benefit from being in this study. You may experience improved health and well-being, individually and as a couple, as well as less avoidance in dealing with cancer, better communication quality, and better coping as a couple. By taking part, you could help us find better ways of supporting breast and gynecologic cancer survivors and their partners/spouses.

**Confidentiality:** Study staff will protect your personal information closely so no one will be able to connect your responses and any other information that identifies you. Directly identifying information (e.g. names, addresses) will be safeguarded and maintained under controlled conditions. Your identifying information will be replaced with codes. Only the research team will have access to information that identifies you to carry out this research study. Any personal information that could identify you will be removed or changed before files are shared with other researchers or results are made public. You will not be identified in any publication or presentation from this study. The security of information collected online cannot be guaranteed.

All sessions will be audio-recorded to monitor how the program is delivered. You should not enroll if you do not wish to be recorded.

We will conduct interviews in a private room where no one can accidentally hear what you say. Your name and what you tell us in the interviews will be kept private to the extent allowed by law. Your responses will be audio-recorded and written out word-for-word. After the interviews, the researchers will listen to the recordings to compare them to the written version to be sure it is accurate. After the analyses are complete, interview recordings will be destroyed.

Regulatory agencies and Oregon State University employees may access or inspect records pertaining to this research as part of routine oversight or university business. Some of these records could contain information that personally identifies you.

A description of this clinical trial will be available on http://www.ClinicalTrials.gov, as required by U.S. Law. This website will not include information that can identify you. At most, the website will include a summary of the results. You can search this website at any time.

**Payment:** You will be paid for being in this research study. Each participant will receive a $10 Amazon gift card ($20 per couple) after completing each online survey. Each participant will receive a $20 Amazon gift card ($40 per couple) after completing a follow-up telephone interview.

**Study contacts:** We would like you to ask us questions if there is anything about the study that you do not understand. You can call us at 541-737-2323 or email us at [Jessica.Gorman@oregonstate.edu](mailto:Jessica.Gorman@oregonstate.edu)

You can also contact the Human Research Protection Program with any concerns that you have about your rights or welfare as a study participant. This office can be reached at (541) 737-8008 or by email at [IRB@oregonstate.edu](mailto:IRB@oregonstate.edu)

**Consent:** By electronically accepting, you tell us that you have gotten all of the information you need; that you have received clear answers to your questions, and that you agree to take part in the research study. You will receive a copy of this form.

Date of the consent

**Future Contact:** We would like your permission to contact you in the future about additions to this study that we may develop or about other research studies that may interest you.

You may agree to the study even if you do not allow us to contact you in the future about other studies. By electronically accepting, you are giving us permission to contact you about other research studies. Yes__ No__
